# Rumen-protected guanidinoacetic acid improves growth performance in beef cattle under chronic heat stress by reshaping gut microbiota and modulating serum metabolism

**DOI:** 10.3389/fmicb.2025.1529596

**Published:** 2025-02-18

**Authors:** Qiang Geng, Wei Lin, Libin Yang, Xiaofei Hu, Xinjun Qiu

**Affiliations:** ^1^School of Tropical Agriculture and Forestry (School of Agricultural and Rural Affairs, School of Rural Revitalization), Hainan University, Haikou, China; ^2^Beijing Gendone Biotechnology Co., Ltd., Beijing, China

**Keywords:** heat stress, beef cattle, guanidinoacetic acid, gut microbiota, serum metabolism

## Abstract

This study aimed to investigate the effects of rumen-protected guanidinoacetic acid (RP-GAA) on growth performance, gut microbiota, and serum metabolism in beef cattle under chronic heat stress. A randomized block design was employed to allocate 14 F1 Simmental crossbred cattle (Simmental ♂ × *Bos indicus* ♀) with an average body weight of 312.5 ± 55.0 kg into two groups (*n* = 7): a control group was fed a basal diet without RP-GAA and a treatment group was fed the same basal diet supplemented with 10.0 g/day of RP-GAA. During feeding experiments, at 2 p.m., the average temperature increased to 31.5°C, with a relative humidity of 69.5% and a THI of 83.2. All animals are under chronic heat stress. The results indicated that RP-GAA supplementation significantly increased dry matter intake and feed conversion ratio in beef cattle under chronic heat stress (*p* < 0.05). RP-GAA supplementation tended to reduce respiratory rate or rectal temperature (*p* < 0.1). Compared to the control group, the treatment group exhibited significantly higher glucose, blood urea nitrogen, total cholesterol, high-density lipoprotein cholesterol, and low-density lipoprotein cholesterol levels (*p* < 0.05). 16S rRNA gene sequencing revealed that RP-GAA supplementation significantly altered the ruminal microbiota composition, increasing the abundance of Firmicutes and Bacteroidota (*p* < 0.05), while reducing Proteobacteria (*p* < 0.01). Principal coordinate analysis (PCoA) and Adonis test (*R*^2^ = 0.190, *p* = 0.003) jointly revealed a distinct difference in fecal microbiota structures between the two groups. Metabolomic analysis identified significant changes in pathways related to creatine synthesis, energy metabolism, and nitrogen utilization, supported by the orthogonal partial least squares discriminant analysis model (*R*^2^*Y* = 0.983, *Q*^2^ = 0.836, *p* < 0.05). These findings suggest that RP-GAAenhances energy homeostasis, supports gut health, and mitigates the adverse effects of heat stress, providing a promising strategy to improve production efficiency and animal welfare in heat-stressed cattle.

## 1 Introduction

Heat stress poses significant challenges to livestock production globally, especially in tropical and subtropical regions where high temperatures and humidity persist for extended periods. Ruminants, pigs and poultry have specific physiological characteristics (Li et al., 2023; Yue et al., 2023; Al-Suwailem et al., 2024; Hussan et al., n.d.), such as rumen fermentation, sweating disorders and feather insulation, along with higher metabolic rates and growth rates, making them more vulnerable to heat stress (Gonzalez-Rivas et al., 2020). At the individual level, heat stress leads to reduced feed intake, growth retardation, intestinal imbalances, decreased reproductive performance, weakened immunity, and endocrine disorders in livestock and poultry (Rostagno, 2020; Zhou et al., 2024; Dahliana and Zulfikhar, n.d.). At the cellular level, heat stress induces oxidative stress, endoplasmic reticulum stress, and neurochemical stress, while impairing protein folding and mitochondrial function, and disrupting the cell cycle (Cao and Kaufman, 2014; Park et al., 2005; Oğuz et al., 2024). In addition, heat stress can also cause oxidative stress, especially the oxidation of lipids and proteins, it is easier for bacterial replication, shorten the shelf life of meat, and threaten food safety (Lara and Rostagno, 2013; Almundarii, 2023). The adverse effect of heat stress has seriously hindered the development of livestock industry all over the world. How to better relieve heat stress of livestock and poultry has become the focus of scientific research and animal husbandry.

Growing evidence suggests that guanidinoacetic acid (GAA) has potential feeding value for alleviating heat stress in livestock and poultry (Zhang et al., 2022). GAA serves as a precursor in the synthesis of creatine, which is crucial for energy metabolism, particularly in muscle cells, by facilitating the storage and transfer of high-energy phosphate groups through the creatine-phosphate system. Several important physiological functions are also served by GAA, including promoting insulin secretion, regulating neurological processes, and mitigating oxidative stress. In studies of broilers, GAA has been shown to enhance the intestinal mucosal immune barrier, conserve arginine, and improve feed conversion efficiency under heat stress (Majdeddin et al., 2020; Peng et al., 2023). In ruminant studies, Non-protected GAA enhances production performance by improving feed intake, crude protein apparent digestibility, rumen microbial protein synthesis and nitrogen deposition in mutton sheep and beef cattle (Li et al., 2020; Ren et al., 2022). The rumen degradation rate of Non-protected GAA was 54–84% (Zhang, 2022), whereas rumen-protected GAA (RP-GAA) demonstrates more stable chemical properties compared to GAA and creatine (Bingrui et al., 2023). However, there are limited research on whether rumen-protect GAA can improve the growth performance of beef cattle under heat stress conditions, and the underlying mechanisms by which GAA alleviates heat stress in beef cattle remain unclear.

Gut microbiota is significantly altered by heat stress, with a reduction in beneficial microbes and an increase in harmful bacteria (Wang et al., 2024). This imbalance compromises intestinal barrier function, heightens inflammation, and disrupts nutrient absorption and immune regulation, negatively affecting animal health and performance under heat stress (Myer et al., 2017). It remains unclear whether RP-GAA can reshape the gut microbiota to counteract the adverse effects of heat stress.

Metabolomics provides a framework for analyzing individuals with specific phenotypes at the molecular level in cell biology, personalized medicine and systems biology (Johnson and Gonzalez, 2012; Kuehnbaum and Britz-McKibbin, 2013; Patti et al., 2012). Metabolites are regarded as the final response of a biological system to environmental changes or gene regulations (Aretz and Meierhofer, 2016), so the metabolome is the most predictive of phenotype (Dettmer et al., 2007; Kuehnbaum and Britz-McKibbin, 2013). Given the central role of creatine metabolism in energy homeostasis and its potential importance under heat stress, metabolomic approaches are essential for assessing the impact of RP-GAA on serum metabolism in cattle.

Therefore, this study investigates the effects of RP-GAA on growth performance, serum metabolism, and gut microbiota in beef cattle under chronic heat stress. This trial holds practical significance for improving beef cattle production efficiency in tropical regions.

## 2 Material and method

The current trail was approved by the Hainan University Animal Ethics Committee (approval number: HNUAUCC-2024-00161).

### 2.1 Experimental animal and management

This feeding experiment was carried out at the beef cattle farm of Xiangyang Agricultural Development Co., Ltd. in Hainan Province, located at 109.339°E longitude and 19.827°N latitude. A randomized block design was employed to allocate 14 F1 Simmental crossbred cattle (Simmental ♂ × *Bos indicus* ♀) with an average body weight of 312.5 ± 55.0 kg into two groups: a control group was fed a basal diet without RP-GAA and a treatment group was fed the same basal diet supplemented with 10.0 g/day of RP-GAA. Each animal was housed in a tie-stall system and fed a total mixed ration twice daily, at 08:00 and 16:00. The nutrient composition and ingredients of the total mixed diet consisted of 33.27% corn, 10.35% soybean meal, 10% wheat bran, 0.92% NaCl, 0.93% NaHCO₃, 0.93% premix, and 43.6% roughages (*Pennisetum × sinese*) on dry matter basis. All animals had ad libitum access to feed and water, with feed residue maintained below 5%. The adaptation period lasted 7 days, followed by a 70-day treatment period (July 24, 2023 to January 11, 2024).

### 2.2 Monitoring of ambient temperature and humidity

Ambient temperature and relative humidity were daily measured and recorded using a GSP-8A Temperature Recorder (Jiangsu Jingchuang Electronics Co., Ltd., Xuzhou). The Temperature-Humidity Index (THI) was calculated using the formula: THI = 0.8tdb + RH (tdb−14.4) + 46.4 (Brown-Brandl, 2018), where tdb is the dry bulb temperature and RH is the relative humidity.

### 2.3 Measurement of rectal temperatures and respiratory rates

From days 32–34 of the trial, rectal temperature of each animal was measured using an electronic thermometer at 40-min intervals from 12:00 to 14:00. Respiration rate was measured via palpation, by placing a hand on the animal’s chest and counting the number of breaths for 10 min at 40-min intervals.

### 2.4 Growth performance assessment

On days 0 and 70 of the trial, each animal was weighed and recorded before morning feeding to calculate average daily gain (ADG). During this period, daily feed intake and feed residue were weighed and recorded to calculate dry matter intake (DMI) and the ratio of gain to feed (feed conversion ration, FCR).

### 2.5 Sample collection procedures

All animals’ rectal feces and blood were sampled on day 43 of the trial. Blood samples were collected from the jugular vein before the morning feeding. The samples were allowed to stand at room temperature for 30 min and then centrifuged at 3500 rpm for 10 min using a centrifuge (TGL-16, Jiangsu) to obtain supernate. Finally, serum samples were stored a refrigerator (Esco Lexicon II UUS-363A-3-SS, Singapore) at −80°C until analyses. Fecal samples were collected at 16:00, then transferred into 2 mL freezing tubes, and finally stored at −80°C until the 16S rRNA gene sequencing.

### 2.6 Measurement of serum biochemical parameters

Serum biochemical parameters, including glucose (GLU), blood urea nitrogen (BUN), creatinine (CRE), uric acid (UA), lactate dehydrogenase (LDH), low-density lipoprotein cholesterol (LDL-C), high-density lipoprotein cholesterol (HDL-C), total cholesterol (TCHO), alanine aminotransferase (ALT), aspartate aminotransferase (AST), gamma-glutamyl transferase (GGT), alkaline phosphatase (ALP), total protein (TP), albumin (ALB), globulin (GLOB), total bilirubin (TBIL), direct bilirubin (DBIL), indirect bilirubin (IBIL), triglycerides (TG), and. (CK) were measured using commercial assay kits from Nanjing Jianchen Bioengineering Co., Ltd. All measurements were performed in duplicate to ensure accuracy and reliability of the results.

### 2.7 16S rRNA gene sequencing for fecal microbial profiling

#### 2.7.1 Fecal genomic DNA extraction

Fecal genomic DNA was extracted using the E.Z.N.A. DNA Kit (Omega Bio-tek Inc., USA), following the manufacturer’s protocol. DNA concentration and purity were determined using a NanoDrop 2000 spectrophotometer (Thermo Scientific Inc., USA). Extracted DNA was stored at −20°C until further analysis.

#### 2.7.2 Amplification of the bacterial 16S rRNA gene

The V3-V4 hypervariable regions of the bacterial 16S rRNA gene were amplified using universal primers 338F (5′-ACTCCTACGGGAGGCAGCAG-3′) and 806R (5′-GGACTACNNGGGTATCTAAT-3′). PCR amplification was performed on an ABI 9700 PCR thermocycler (Applied Biosystems, USA) under the following conditions: an initial denaturation at 95°C for 5 min, followed by 28 cycles of 95°C for 45 s, 55°C for 50 s, and 72°C for 45 s, with a final extension at 72°C for 10 min.

#### 2.7.3 PCR product purification and 16S rRNA library preparation

Following PCR amplification, the products were purified using the Agencourt AMPure XP Kit (Beckman Coulter Inc., USA). Sequencing libraries were constructed using the NEB Next Ultra II DNA Library Prep Kit (New England Biolabs Inc., USA) according to the manufacturer’s instructions. The quality and concentration of the libraries were verified using a NanoDrop 2000 (ThermoFisher Scientific Inc., USA), an Agilent 2100 Bioanalyzer (Agilent Technologies Inc., USA), and an ABI StepOnePlus Real-Time PCR System (Applied Biosystems Inc., USA).

#### 2.7.4 High-throughput 16S rRNA gene sequencing

Sequencing of the 16S rRNA libraries was carried out on the Illumina MiSeq platform (Illumina Inc., USA) at Beijing Allwegene Technology Co., Ltd. Post-sequencing image analysis, base calling, and error estimation were processed using the Illumina Analysis Pipeline Version 2.6 (Illumina Inc., USA). Taxonomic classification was conducted using Silva 138 database.

#### 2.7.5 Sequencing data preprocessing and quality control

Raw sequencing data were stored in FASTQ format and demultiplexed into individual samples based on barcode sequences. Quality control was performed using Pear (v0.9.6), removing ambiguous bases, primer mismatches, and trimming bases with a quality score below Q20. Paired-end reads were merged with a minimum overlap of 10 bp and a *p*-value of 0.0001 to generate Fasta sequences. Chimeric sequences were removed using the uchime method in Vsearch (v2.7.1) for known databases, and the denovo method for unknown databases. Short sequences not meeting length requirements were also filtered out.

#### 2.7.6 Operational taxonomic units (OTU) clustering and taxonomic annotation

After quality control, high-quality sequences were clustered into OTUs using the Uparse method with a 97% similarity threshold, utilizing Vsearch (v2.13.3) for the clustering and QIIME (v1.8.0) for overall analysis management. For taxonomic annotation, sequences were matched against the Silva 138 database.^1^

#### 2.7.7 Dominant bacterial taxa

Differences in bacterial composition between the treatment and control groups were analyzed at the phylum and genus levels. Phyla with relative abundances >0.1% were defined as dominant phyla, and genera with relative abundances >1% were classified as dominant genera.

#### 2.7.8 Bacterial *α*-diversity analysis

The OTU tables were rarefied to the minimum sequence depth across all samples to ensure comparability. Alpha diversity, including Chao1, observed species, PD whole tree, and Shannon index, was assessed using QIIME (v1.8.0).

#### 2.7.9 Principal coordinates analysis (PCoA) and Adonis test

To explore sample similarities and differences in bacterial community composition between the two groups, PCoA was used for dimensionality reduction with the Bray-Curtis distance metric. Furthermore, Adonis test was performed to statistically validate the significance of the observed differences. Both analyses were performed using the vegan package (v2.6.4) in R (v4.3.1).

#### 2.7.10 Linear discriminant analysis effect size (LEfSe)

Non-parametric Kruskal-Wallis test was first used to identify species with significant abundance differences between groups, with a threshold of 0.05. Significant species from this analysis were then further examined using paired Wilcoxon rank-sum test, with a threshold of 0.05. Finally, Linear Discriminant Analysis (LDA) was employed for dimensionality reduction and to assess the impact of significant species (LDA score), with a threshold of 3. All analyses and data visualization were performed using Python (v3.12.0) and relevant libraries.

### 2.8 Ultra high performance liquid chromatography-mass spectrometry (UHPLC–MS) analysis

#### 2.8.1 Extraction of serum metabolites

Transfer 50 μL of serum into an EP tube, and add 200 μL of extraction solvent (methanol: acetonitrile, 1:1, v/v) containing isotopically labeled internal standards. Vortex the mixture for 30 s using a Waltham homogenizer (Shanghai Jingxin Technology Co., Ltd.), followed by sonication for 10 min with an ultrasonic processor (PS-60AL, Shenzhen Leidibang Electronics Co., Ltd.) in an ice-water bath. Incubate the sample at −40°C for 1 h to facilitate metabolite precipitation. Centrifuge at 4°C, 12,000 rpm (13,800 × *g*, radius 8.6 cm) for 15 min to separate the supernatant. Transfer the supernatant to a sample vial for subsequent analysis. Prepare a quality control (QC) sample by combining equal volumes of supernatant from all individual samples.

#### 2.8.2 UHPLC–MS instrumentation and methodology

Serum metabolites were analyzed using a Vanquish UHPLC system (Thermo Fisher Scientific). Chromatographic separation was performed on a Waters ACQUITY UPLC BEH Amide column (2.1 mm × 50 mm, 1.7 μm). Mobile phase A consisted of water with 25 mmol/L ammonium acetate and 25 mmol/L ammonia, while mobile phase B was acetonitrile. The sample tray was maintained at 4°C, and the injection volume was set at 2 μL. Mass spectrometry (MS1 and MS2) data were acquired using the Orbitrap Exploris 120 mass spectrometer, controlled by Xcalibur software (version 4.4, Thermo Fisher). The instrument parameters were as follows: Sheath gas flow rate: 50 Arb; Auxiliary gas flow rate: 15 Arb; Capillary temperature: 320°C; Full MS resolution: 60,000; MS/MS resolution: 15,000; Collision energy: stepped normalized collision energy (SNCE) 20/30/40; Spray voltage: 3.8 kV (positive mode) or −3.4 kV (negative mode).

#### 2.8.3 Metabolite identification and quality control

Raw data were converted to mzXML format using ProteoWizard software, and metabolite identification was performed using the BiotreeDB database (v3.0). To minimize systematic error and enhance the biological significance of the results, we applied a series of data management steps to the raw data. This included outlier filtering, where peaks were filtered based on noise and relative standard deviation (RSD, or coefficient of variation, CV). Missing value filtering was performed by excluding peaks with more than 50% missing values within a single group or across all groups. Missing values were imputed using half of the minimum observed value. Finally, data were normalized using internal standards (IS). To ensure the stability of the analytical method, variations in the response peak heights of internal standards across QC samples were examined, and blank samples interspersed throughout the experiment were used to monitor potential analyte carryover during the detection process.

#### 2.8.4 Principal component analysis (PCA) and Adonis test

To reduce the dimensionality of the data, the original variables were transformed into a set of orthogonal components through PCA. The data were standardized before analysis, and the process was conducted using the prcomp function from the stats (v4.4.1) package in R. Additionally, the Adonis test was performed to statistically validate the observed group differences, following the methods described above.

#### 2.8.5 Orthogonal partial least squares discriminant analysis (OPLS-DA) and permutation test validation

In the OPLS-DA, the model aims to maximize the separation between groups and identify variables contributing to classification. Prior to analysis, data were scaled using unit variance scaling. The OPLS-DA model was constructed to highlight systematic variations related to group classification while removing orthogonal variations unrelated to class differences. Model validation was performed through 200 permutation tests and cross-validation to assess robustness and avoid overfitting. Analyses were conducted using the ropls package (v1.4.2) in R.

#### 2.8.6 Screening criteria for differential metabolites

The selection of differential metabolites was based on the following two criteria: first, a Student’s t-test was conducted to evaluate the differences in metabolites between groups, requiring a *p*-value of less than 0.05. Second, the Variable Importance in the Projection (VIP) for each metabolite was calculated from the OPLS-DA model, with metabolites having a VIP value greater than 1 being selected. These two criteria were used in combination to ensure that the selected metabolites exhibited statistically significant differences and made important contributions to group classification.

#### 2.8.7 Kyoto encyclopedia of genes and genomes (KEGG) enrichment analysis of differential metabolites

KEGG enrichment analysis was performed to assess whether specific metabolic pathways related to the differential metabolites were significantly enriched. Differential metabolites were mapped to their corresponding pathways using the KEGG database.^2^ The significance of pathway enrichment was determined using Fisher’s exact test, which compared the proportion of differential metabolites in each pathway against the expected proportion based on all detected metabolites. Pathways with a *p*-value less than 0.05 were considered significantly enriched. The Database for Annotation (DA) score was used to reflect the overall differential abundance of metabolites within each pathway.

### 2.9 Statistical analysis and data visualization

Repeated measures Analysis of Variance (ANOVA) was employed to analyze the effects of RP-GAA supplementation on physiological parameters, growth performance, and serum biochemical parameters. The model can be expressed as *y = Treat + Block*, where “Treat” represents the Control group and the RP-GAA group, and “Block” is based on body weight categories. A significance level of *p* < 0.05 was applied to determine statistical significance. Prior to the analysis, normality and homogeneity of variance were assessed. The analyses described above were conducted using R. All figures were generated using the ggplot2 package (v3.4.3), except for the boxplots created with GraphPad Prism (v9.7) and the pathway illustrations related to GAA metabolism, which were generated using WPS software (v12.1.0).

### 2.10 Statistical analysis

Growth performance metrics were modeled using Analysis of Variance for Randomized Block Design, the formula: y = Treat + Block. Other results were analyzed using multifactor ANOVA in IBM SPSS Statistics (version R26.0.0.0) and t-tests in SPSS software.

For visualization, box plots were generated using GraphPad Prism (version 8.3.0.538), while all other plots were created using R (version 3.6.0).

## 3 Results

### 3.1 Ambient temperature, relative humidity and THI

**Figure 1:**
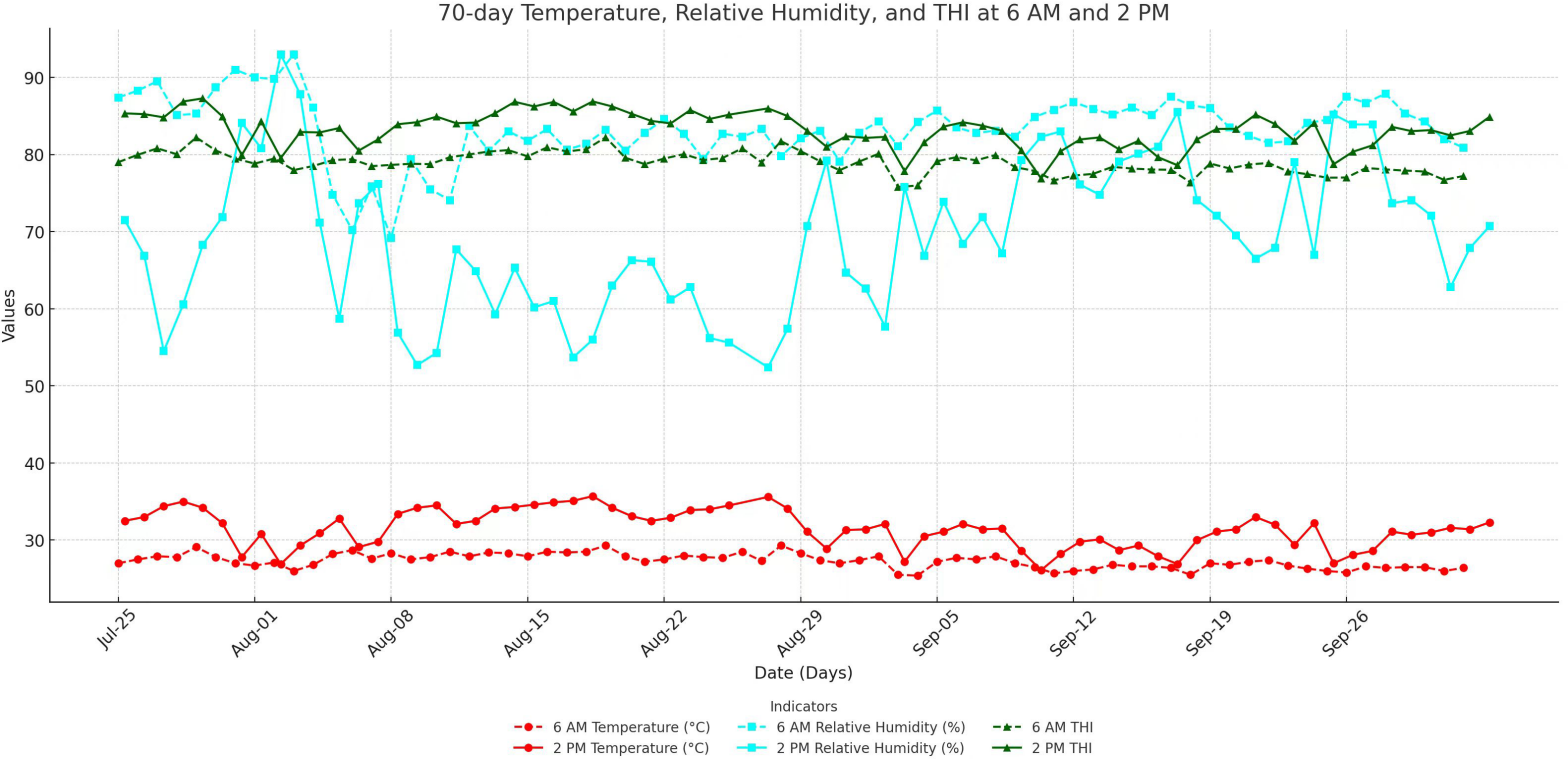
Variation curves of THI and its parameters over the first 70 days of the experiment.

Figure 1 shows the variation curves of THI and its parameters during the first 70 days of the experiment. The average temperature at 6 a.m. was 27.3°C, with a relative humidity of 83.4%, resulting in a THI of 78.9. At 2 p.m., the average temperature increased to 31.5°C, with a relative humidity of 69.5% and a THI of 83.2.

### 3.2 Rectal temperature and respiratory rate

**Figure 2:**
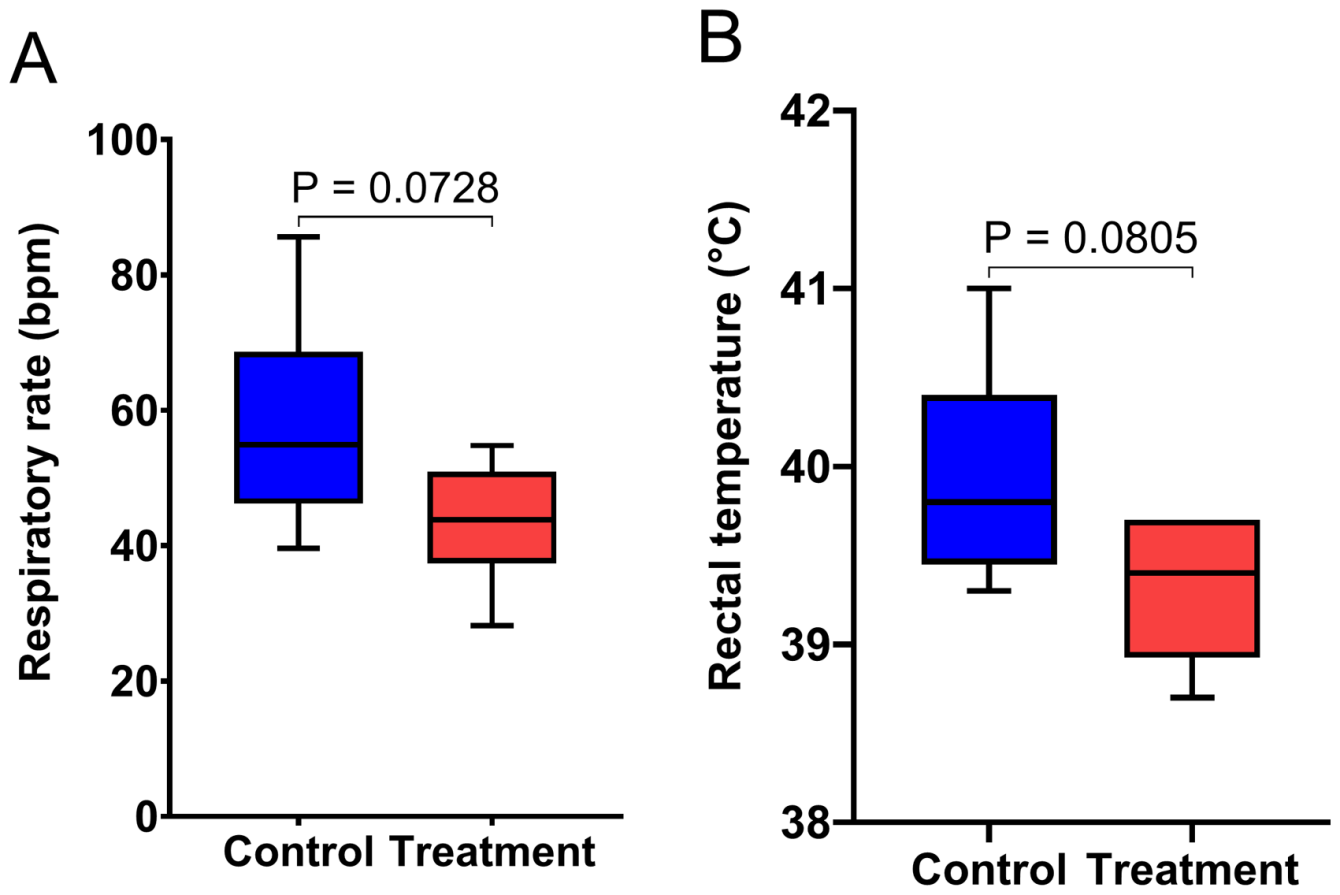
Effect of RP-GAA supplementation on respiratory rate **(A)** and rectal temperature **(B)** of beef cattle under heat stress.

The results of rectal temperature and respiratory rate are presented in Figure 2. Supplementation with RP-GAA tended to deceased the respiratory rate and rectal temperature in beef cattle experiencing chronic heat stress (*p* < 0.1).

### 3.3 Growth performance

**Figure 3:**
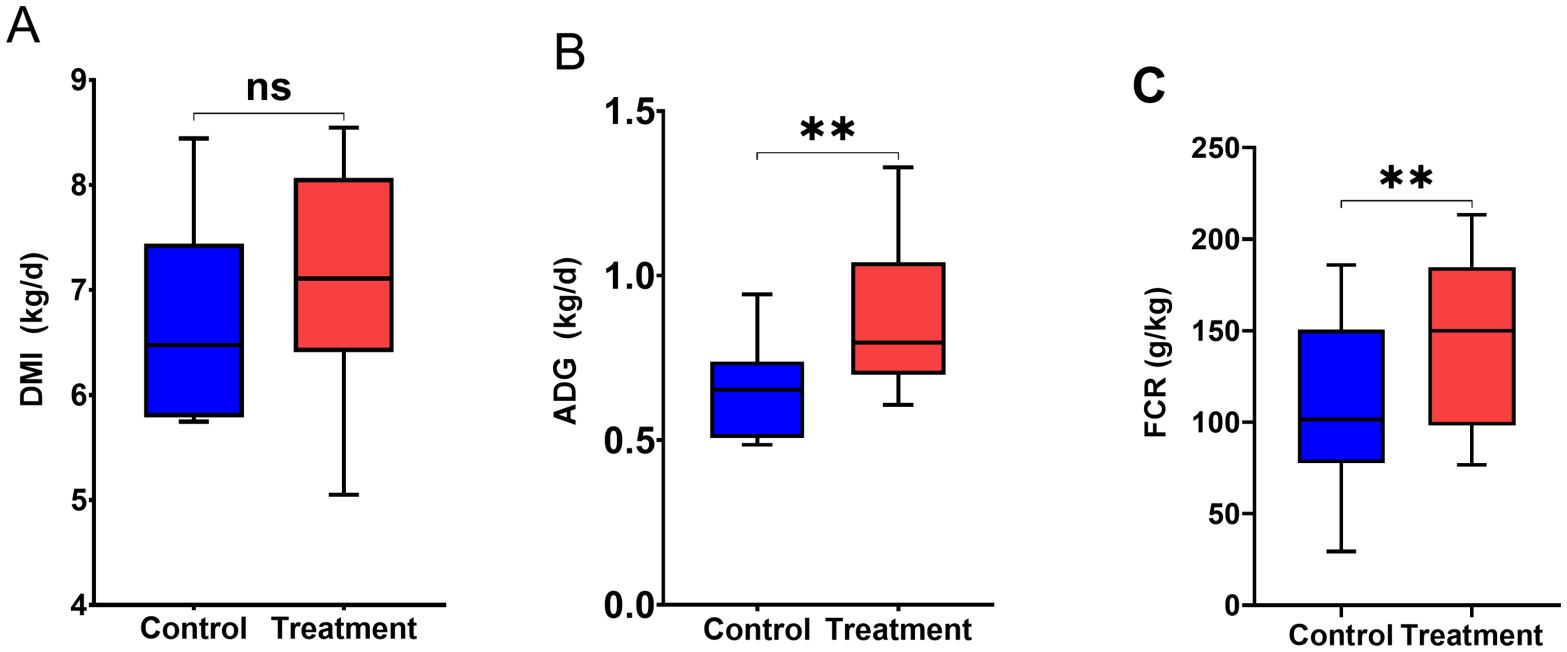
Growth performance including dry matter intake (DMI) **(A)**, average daily gain (ADG) **(B)** and feed conversion ratio (FCR) **(C)** of beef cattle under heat stress with and without RP-GAA supplementation.

Figure 3 illustrates the effect of RP-GAA on the growth performance of beef cattle under chronic heat stress. While RP-GAA supplementation did not had an impact on DMI (*p* < 0.05), but significantly increased ADG and FCR in beef cattle (*p* < 0.05).

### 3.4 Serum biochemical parameters

The serum biochemical results (Table 1) showed that the RP-GAA supplementation group had significantly higher levels of GLU and BUN compared to the control group (*p* < 0.05). Additionally, TCHOI, HDL-C, and LDL-C were significantly elevated in the RP-GAA group (*p* < 0.05).

**Table 1:** Serum biochemical parameters in beef cattle supplemented with RP-GAA under heat stress.

Item^1^	Control	Treatment	SEM	*p*-value
GLU, mmol/L	2.04	2.47	0.126	0.041
BUN, mmol/L	0.85	1.63	0.201	0.043
CRE, umol/L	121	137	5.32	0.144
UA, umol/L	75.7	61.9	5.64	0.104
TG, mmol/L	0.234	0.299	0.032	0.299
TCHOI, mmol/L	1.91	2.54	0.091	0.001
HDL-C, mmol/L	1.18	1.49	0.043	0.003
LDL-C, mmol/L	0.627	0.913	43.4	0.007
ALT, U/L	29.6	29.3	0.058	0.942
AST, U/L	51.4	50.7	2.78	0.903
GGT, U/L	21.3	21.3	4.06	1
ALP, U/L	98.6	131	0.753	0.123
TP, g/L	69.9	70.5	17.2	0.779
ALB, g/L	34.6	34.7	2.86	0.949
GLOB, g/L	35.3	35.8	1.1	0.735
LDH, U/L	782	853	2.95	0.426
CK, U/L	107	106	16.8	0.977

1 GLU, Glucose; BUN, Blood Urea Nitrogen; CRE, Creatinine; UA, Uric Acid; LDH, Lactate Dehydrogenase; LDL-C, Low-Density Lipoprotein Cholesterol; HDL-C, High-Density Lipoprotein Cholesterol; TCHOI, Total Cholesterol; ALT, Alanine Aminotransferase; AST, Aspartate Aminotransferase; GGT, Gamma-Glutamyl Transferase; ALP, Alkaline Phosphatase; TP, Total Protein; ALB, Albumin; GLOB, Globulin; TBIL, Total Bilirubin; DBIL, Direct Bilirubin; IBIL, Indirect Bilirubin; TG, Triglycerides; CK, Creatine Kinase.

### 3.5 16S rRNA gene sequencing for fecal microbial profiling

#### 3.5.1 Quality control and OTU clustering

After the removal of barcode and primer sequences and subsequent quality control, the FASTQ data yielded an average of 72,690 ± 5,100 raw tags per sample. Further filtering steps, including the removal of chimeras and short sequences, produced an average of 65,301 ± 4,587 high-quality clean tags per sample. Upon rarefaction, the number of final tags was standardized to 48,610 across all samples. Clustering these clean tags initially resulted in 9,083 OTUs, which were later reduced to 9,043 OTUs post-rarefaction, with an average of 3,536 ± 185 OTUs per sample.

#### 3.5.2 Dominant bacterial taxa

**Figure 4:**
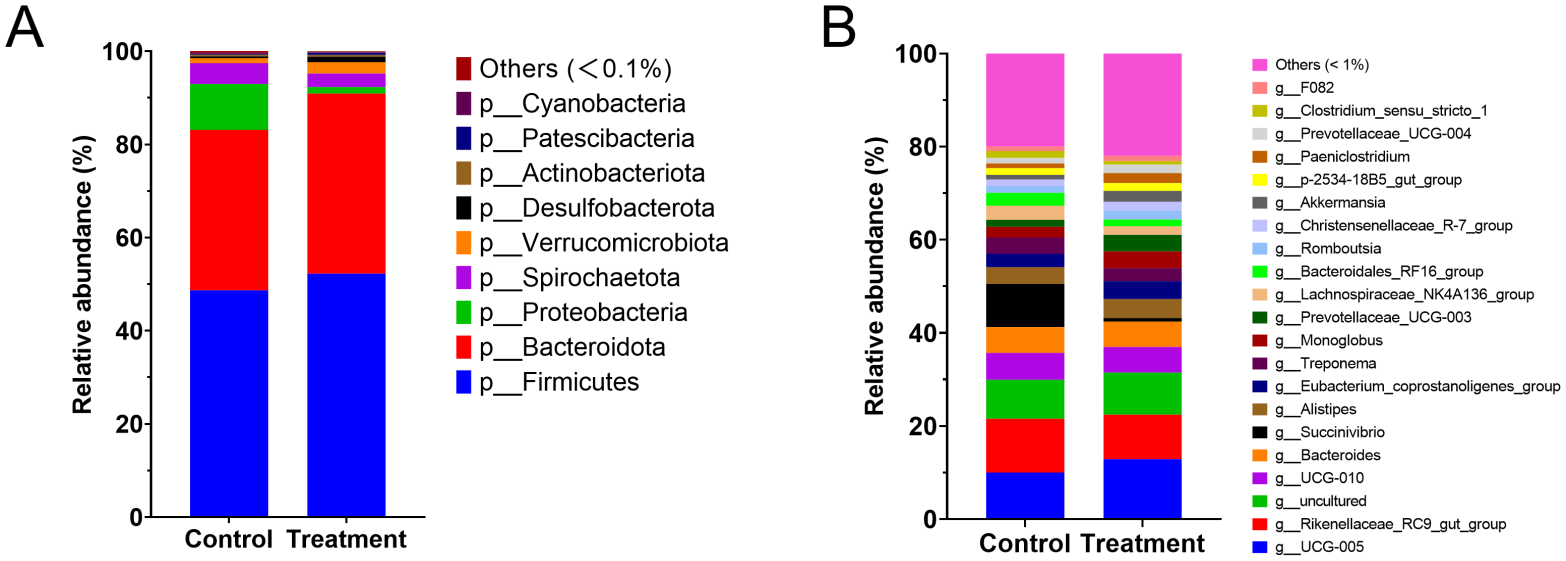
Impact of RP-GAA supplementation on dominant bacterial phyla **(A)** and genera **(B)** in fecal microbiota.

The heatmap (Figure 4) illustrates the effect of RP-GAA supplementation on the dominant bacterial phyla (relative abundance >0.1%) and genera (relative abundance >1%) in the fecal samples. At the phylum level, RP-GAA supplementation led to significant changes in the relative abundance of dominant bacterial phyla. Firmicutes increased in the treatment group compared to the control group (52.3% vs. 48.7%, *p* = 0.041), while Bacteroidota also increased (38.7% vs. 34.5%, *p* = 0.038). In contrast, Proteobacteria decreased significantly in the treatment group (1.4% vs. 9.9%, *p* = 0.002).

At the genus level, RP-GAA supplementation similarly resulted in notable changes in the relative abundance of several bacterial genera. *g__UCG-005* increased in the treatment group compared to the control group (12.89% vs. 10.01%, *p* = 0.045). In contrast, *g__Rikenellaceae_RC9_gut_group* decreased in the treatment group (9.55% vs. 11.58%, *p* = 0.033), as did *g__Succinivibrio* (0.78% vs. 9.29%, *p* = 0.001).

#### 3.5.3 Bacterial α-diversity analysis

**Figure 5:**
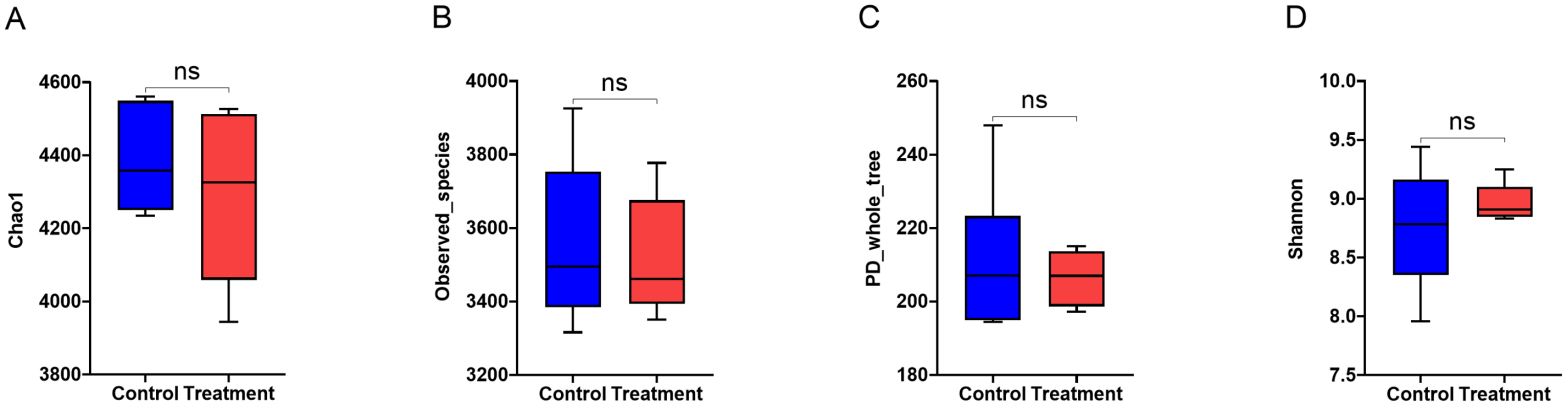
Alpha diversity indices of fecal microbiota in beef cattle supplemented with rumen-protected guanidinoacetic acid (RP-GAA) under heat stress including chao1 **(A)**, observed_species **(B)**, PD_whole_tree **(C)** and shannon **(D)**.

RP-GAA supplementation had no significant effect on any of the alpha diversity indices of the fecal microbiota, including Chao1, observed species, PD whole tree, and Shannon index (*p* > 0.05, Figure 5).

#### 3.5.4 PCoA and Adonis test

**Figure 6:**
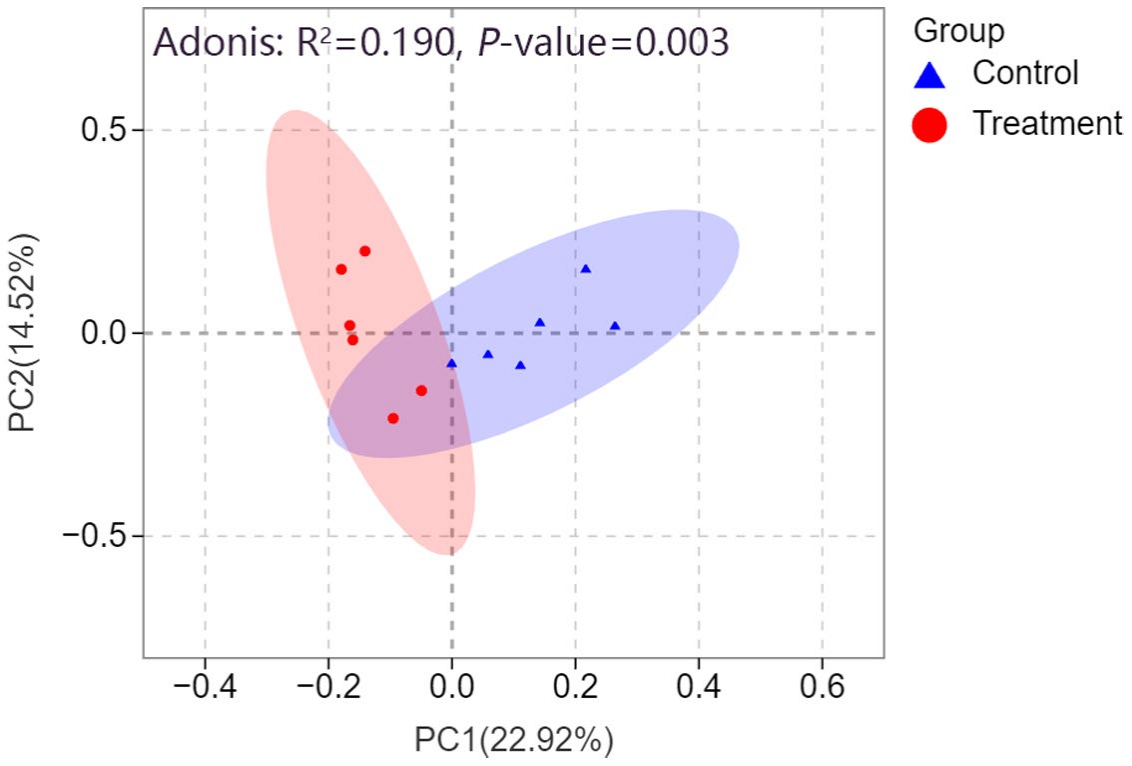
Principal coordinate analysis (PCoA) based on Bray-Curtis distance reveals a clear separation in fecal microbiota structure between the control (blue triangles) and rumen-protected guanidinoacetic acid (RP-GAA) treatment groups (red circles), with 95% confidence intervals represented by ellipses for each group. The Adonis test further confirms the significant difference in microbial community composition between the groups (*R*^2^ = 0.190, *p* = 0.003).

As illustrated in Figure 6, PCoA based on the Bray-Curtis distance metric revealed distinct clustering patterns between the control and treatment groups. PC1 accounted for 24.5% of the variance, while PC2 explained 19%, with the two principal coordinates together capturing 43.5% of the total variance. The treatment group (red circles) and control group (blue triangle) exhibited clear separation, suggesting that RP-GAA supplementation had a significant effect on the microbial community composition. This finding was further corroborated by the Adonis test, which revealed a significant difference between the groups (*R*^2^ = 0.231, *p* = 0.001), explaining 23.1% of the variation in microbial composition.

#### 3.5.5 Linear discriminant analysis effect size (LEfSe)

**Figure 7:**
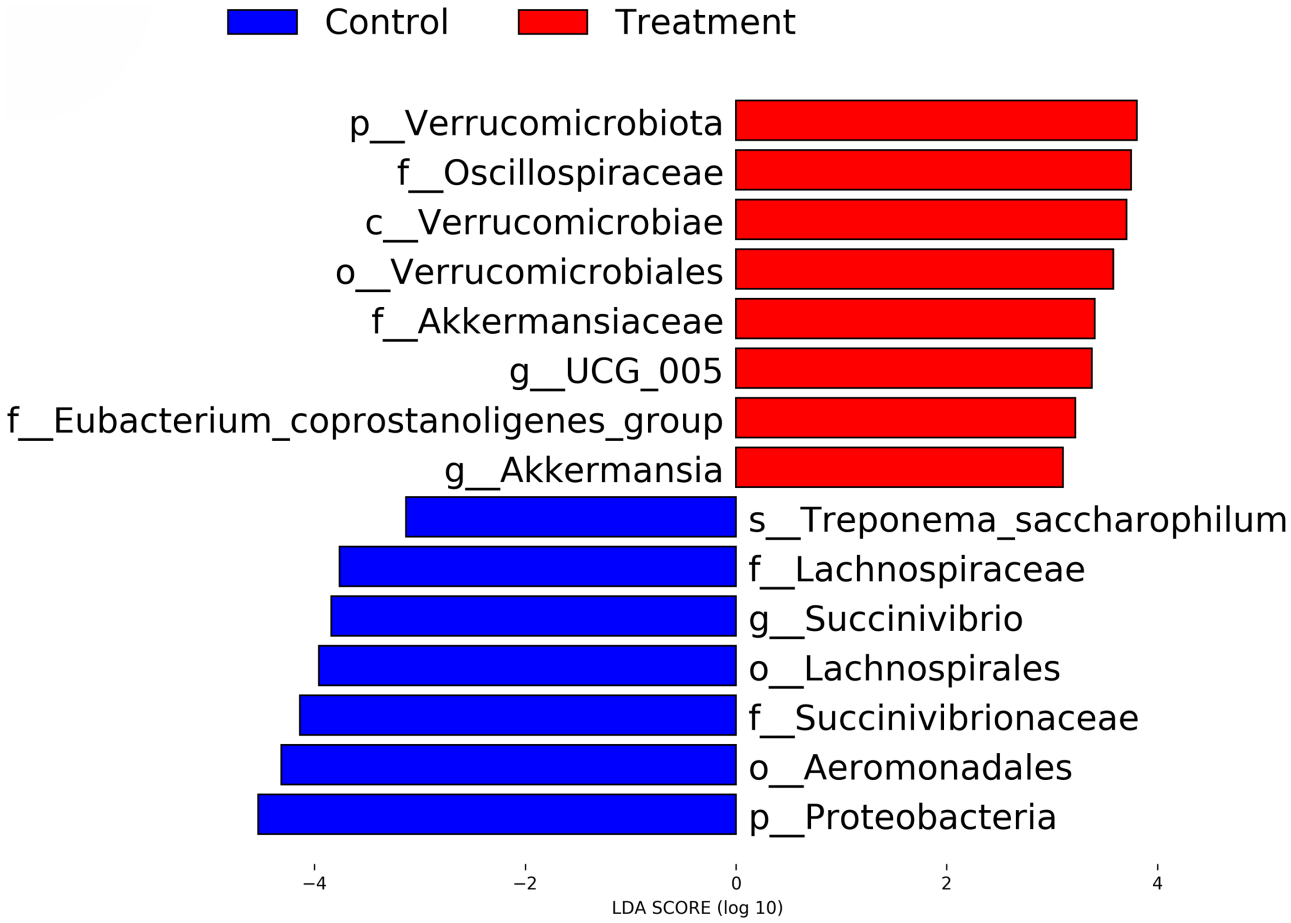
LEfSe analysis identifying differential gut microbial taxa between control and RP-GAA supplemented groups. This plot represents the results of linear discriminant analysis effect size (LEfSe) analysis, highlighting microbial taxa that are differentially abundant between the control (blue) and RP-GAA treatment groups (red). Linear discriminant analysis (LDA) scores greater than 3.0 indicate the most significant taxa driving differences in the gut microbiota composition between the groups.

Using LEfSe analysis, several taxa were identified as significantly different between the Control and Treatment groups. Taxa enriched in the Treatment group included *Verrucomicrobiota, Akkermansia,* and *Oscillospiraceae,* while *Treponema saccharophilum, Lachnospiraceae,* and *Proteobacteria* were more abundant in the Control group. The LDA scores of these taxa, as illustrated in Figure 7, indicate their relative importance, with all significant taxa having an LDA score greater than 3. In the treatment group, *p_Verrucomicrobiota, f_Oscillospiraceae, c_Verrucomicrobiae, o_Verrucomicrobiales, f_Akkermansiaceae, g_UCG_005, f_Eubacterium_coprostanoligenes_group and g_Akkermansia* are positive. In the control group, *s_Treponema_saccharophilum, f_Lachnospiraceae, g_Succinivibrio, o_Lachnospirales, f_Succinivibrionaceae, o_Aeromonadales and p_Proteobacteria* are negative.

### 3.6 Ultra high performance liquid chromatography-mass spectrometry (UHPLC–MS) analysis

#### 3.6.1 Metabolite identification and annotation

After data preprocessing, 10,600 peaks were retained. All identified metabolites were matched against the BiotreeDB database, resulting in the identification of 1,123 metabolites, including 318 KEGG compounds.

#### 3.6.2 PCA and Adonis test

**Figure 8:**
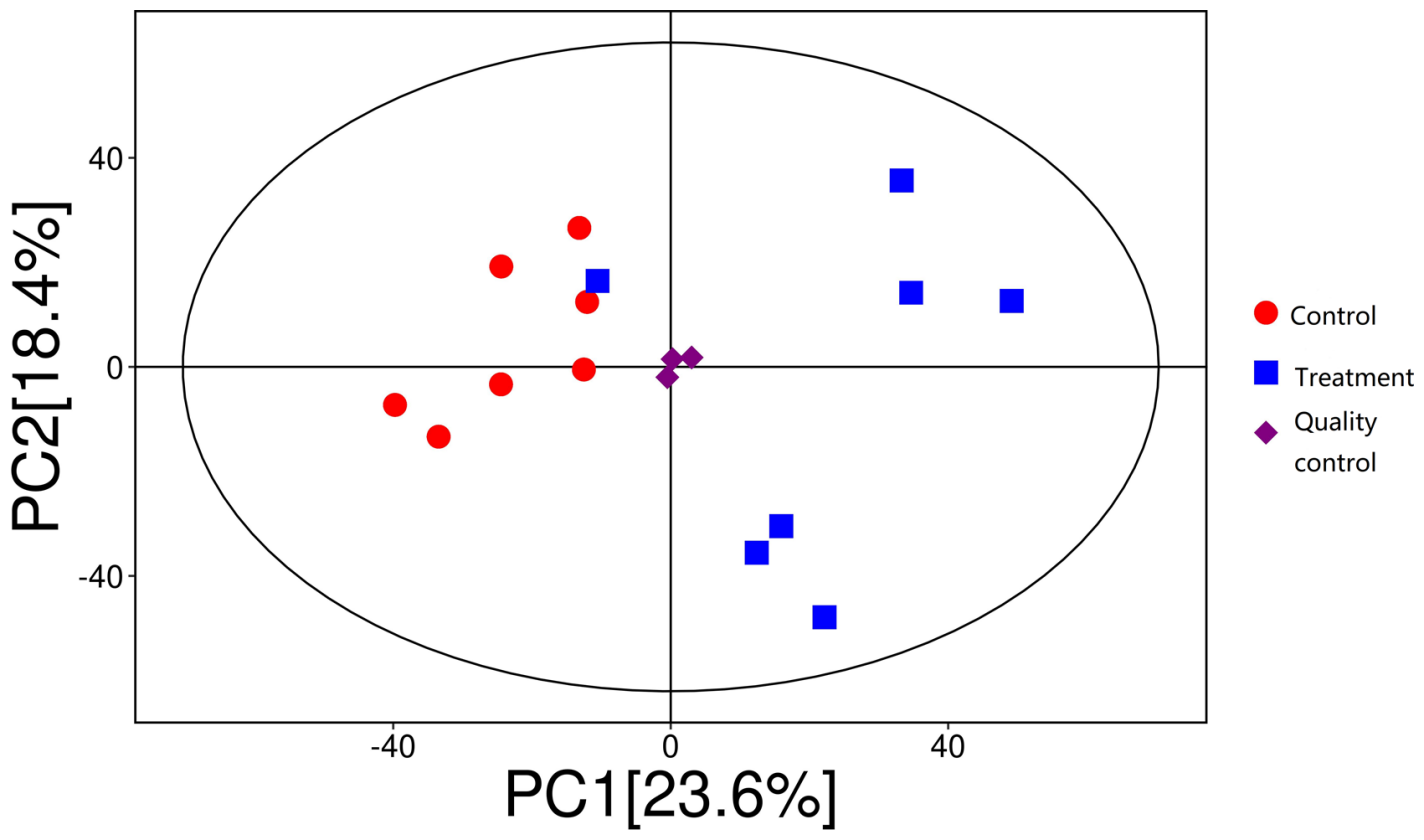
Principal component analysis (PCA) showing distinct clustering of metabolic profiles between control (red circles) and rumen-protected guanidinoacetic acid (RP-GAA) supplemented groups (blue squares), with PC1 and PC2 explaining 42.0% of total variance.

As shown in Figure 8, PCA revealed distinct clustering patterns between the control and treatment groups. PC1 accounted for 18.4% of the variance, while PC2 explained 23.6%, with the two principal components together capturing 42.0% of the total variance. The treatment group (blue squares) and control group (red circles) exhibited clear separation, indicating that RP-GAA supplementation had a significant impact on metabolic composition. This finding was further supported by the Adonis test, which demonstrated a significant difference between the groups, explaining 23.1% of the variation in metabolic composition.

#### 3.6.3 OPLS-DA and permutation test validation

**Figure 9:**
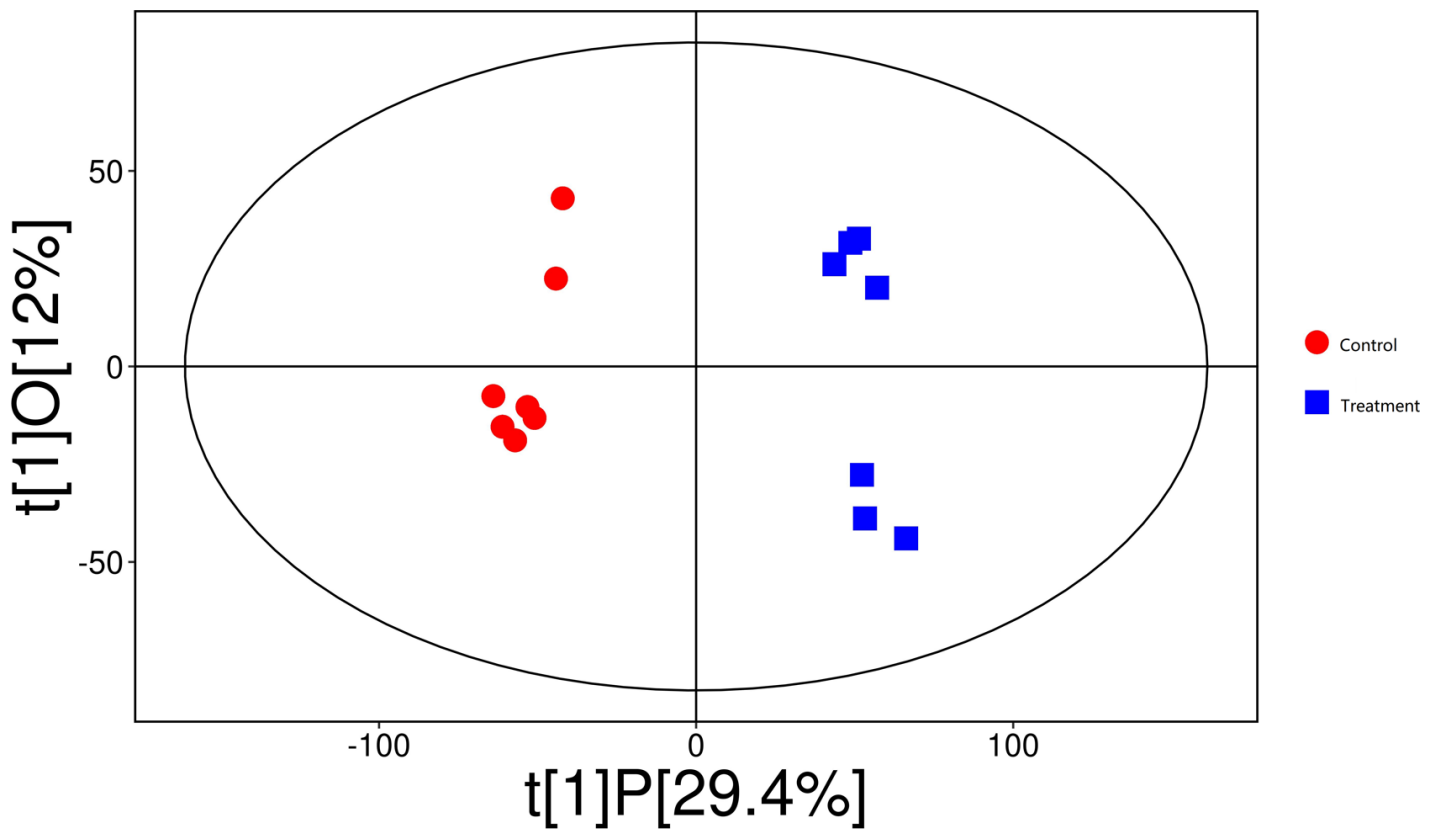
The orthogonal partial least squares discriminant analysis (OPLS-DA) score plot demonstrates distinct clustering patterns between control (red circles) and rumen-protected guanidinoacetic acid (RP-GAA) supplemented groups (blue squares), suggesting that the between-group variation is larger than within-group variation.

**Figure 10:**
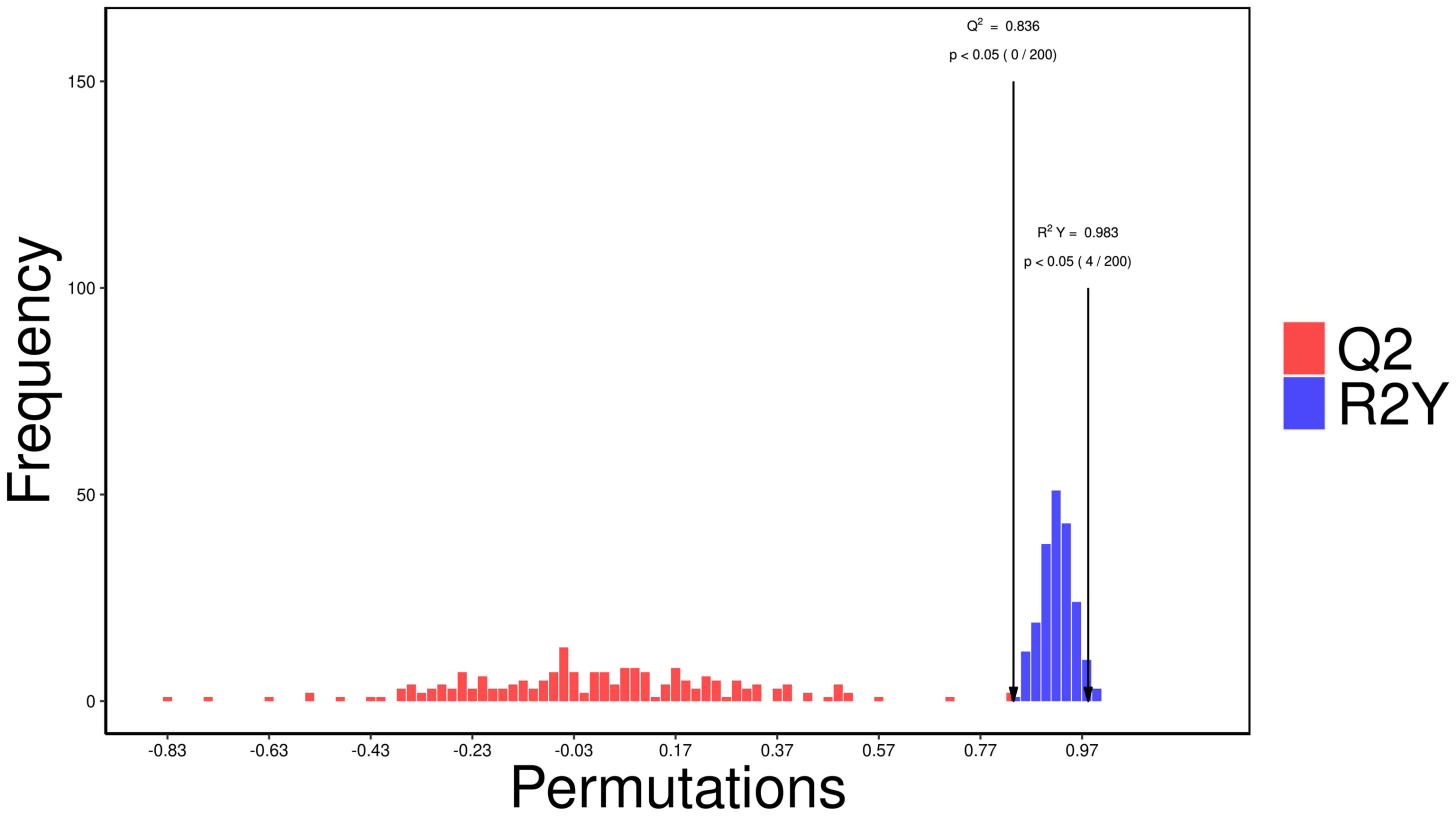
Permutation test validating the orthogonal partial least squares discriminant analysis (OPLS-DA) model of metabolic profiles in control and rumen-protected guanidinoacetic acid (RP-GAA) groups, based on 200 permutations, with an R^2^Y of 0.983 (*p* < 0.05) and a Q^2^ of 0.836 (*p* < 0.05), indicating strong model robustness and performance.

The OPLS-DA score plot (Figure 9) demonstrated distinct clustering patterns between the control and treatment groups, suggesting that the between-group variation is larger than within-group variation. A permutation test (Figure 10) validated the model, with an *R*^2^Y of 0.983 (*p* < 0.05) and a *Q*^2^ of 0.836 (*p* < 0.05), indicating strong model performance and robustness.

#### 3.6.4 Screening of differential metabolites

**Figure 11:**
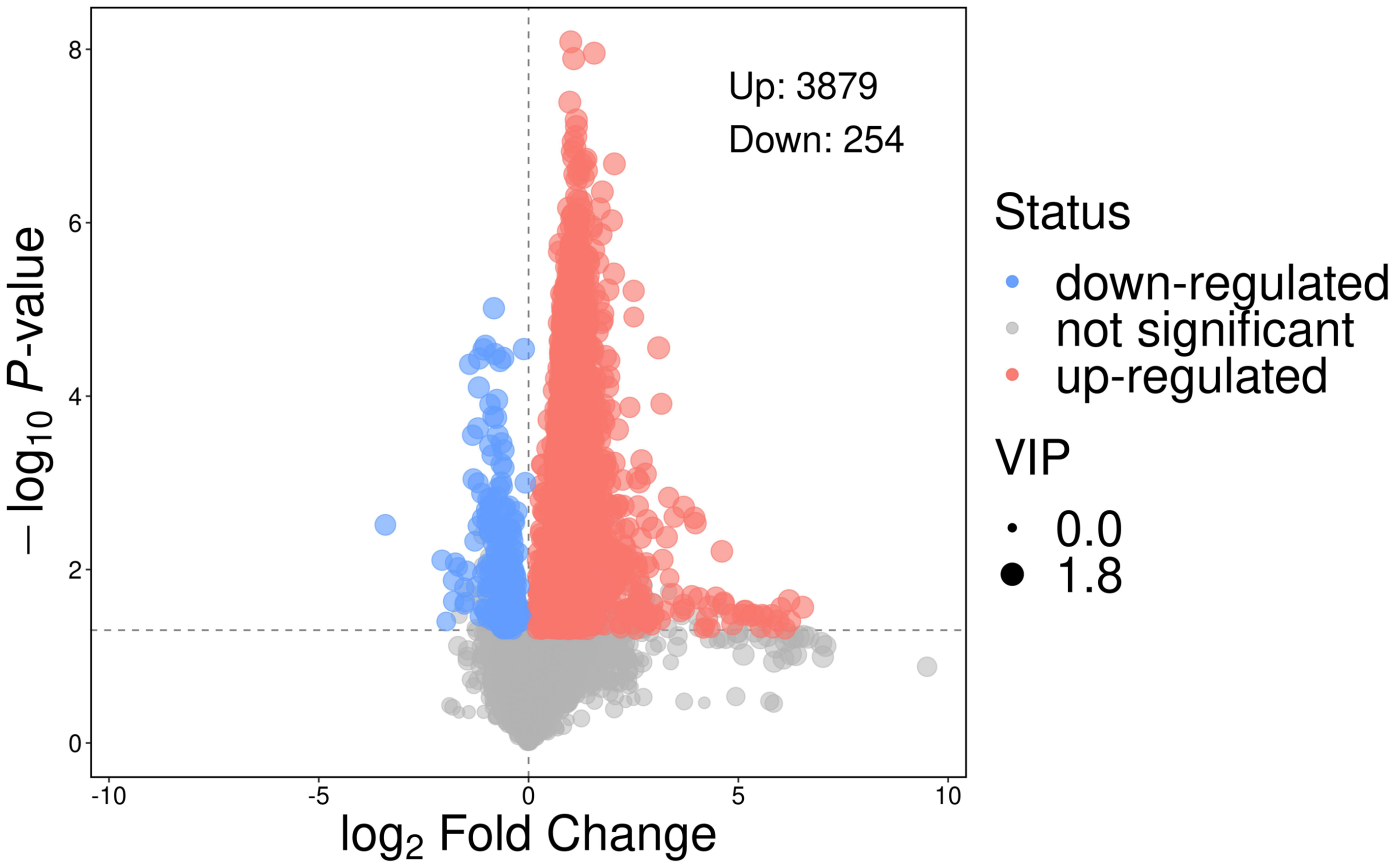
Volcano plot illustrates the differential abundance of metabolites between the control treatment groups, based on t-test and Variable Importance in the Projection (VIP) screening of relative peak intensities. A total of 3,879 metabolites were significantly up-regulated, while 254 metabolites were down-regulated (*p* < 0.05, VIP > 3).

The volcano plot (Figure 11) illustrates the differential abundance of metabolites between the control and treatment groups, based on t-test and VIP screening of relative peak intensities. A total of 3,879 metabolites were significantly up-regulated, while 254 metabolites were down-regulated (*p* < 0.05, VIP > 3).

#### 3.6.5 KEGG enrichment analysis of differential metabolites

**Figure 12:**
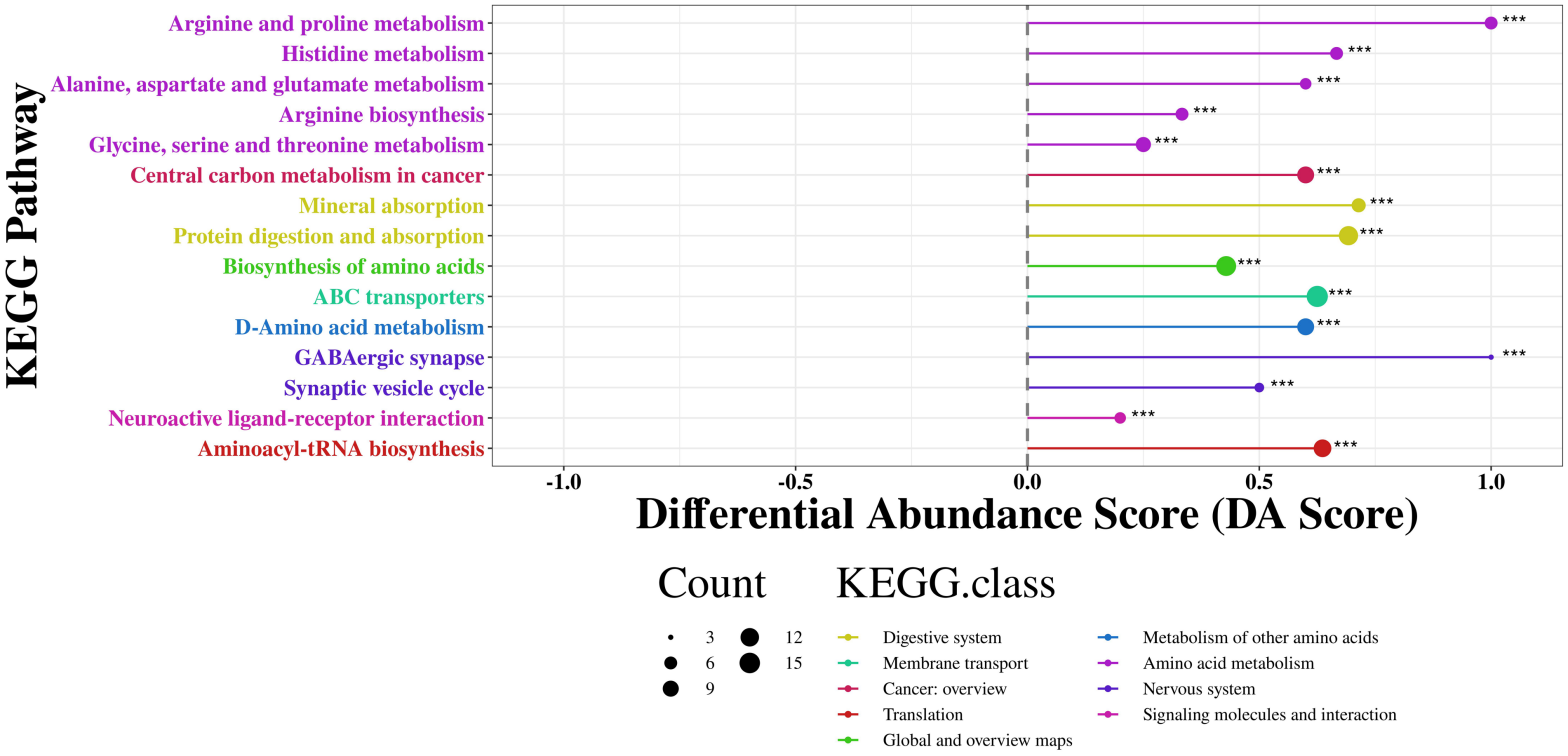
KEGG pathway enrichment analysis of differential metabolites between control and RP-GAA treatment groups, visualized by differential abundance score (DA score).

As shown in the Figure 12, the differential metabolites were primarily enriched in energy metabolism-related pathways such as arginine and proline metabolism, histidine metabolism, alanine, aspartate, and glutamate metabolism, arginine biosynthesis, and glycine, serine, and threonine metabolism (DA Score > 0.5, *p* < 0.001). Additionally, RP-GAA modulated nutrient absorption-related pathways such as protein digestion and absorption and mineral absorption (DA Score > 0.5, *p* < 0.001), as well as neural regulation pathways including GABAergic synapse, synaptic vesicle cycle, and neuroactive ligand-receptor interaction (DA Score > 0.5, *p* < 0.001).

#### 3.6.6 Pathway of creatine synthesis and metabolism involving GAA

**Figure 13:**
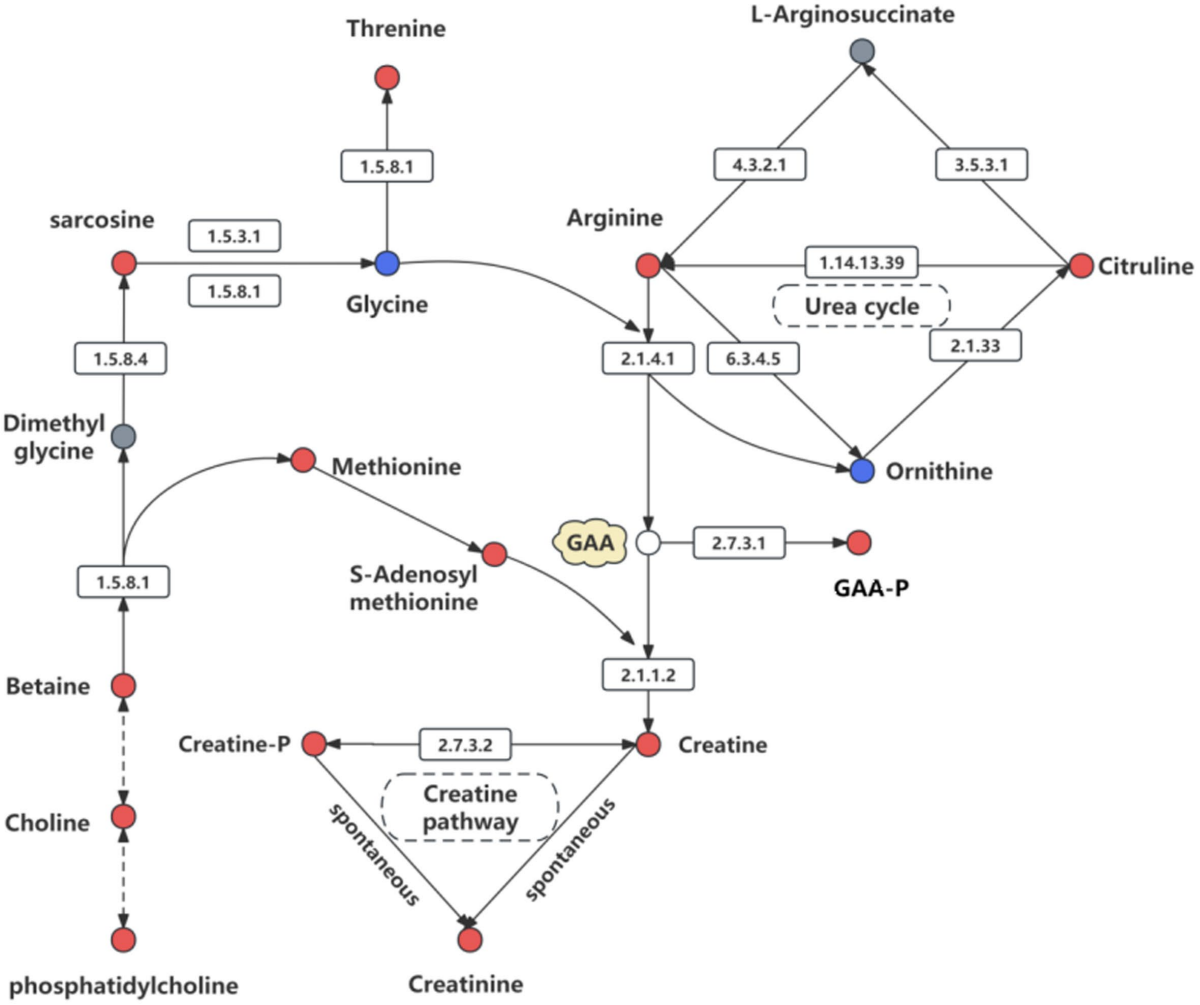
Pathway of creatine synthesis and metabolism involving guanidinoacetic acid (GAA), highlighting key metabolites in the urea cycle, methylation pathway, and creatine phosphate pathway. Red indicates metabolites that are increased, blue represents significantly decreased metabolites, and white denotes metabolites that were not detected.

Figure 13 illustrates the impact of RP-GAA supplementation on metabolic pathways, particularly those involved in creatine synthesis and related processes. The data show that RP-GAA increased the levels of key metabolites, such as GAA and creatine phosphate, which are essential for energy storage in muscle tissue. Furthermore, RP-GAA induced notable changes in the urea cycle and the methylation pathway, suggesting a broader effect on amino acid metabolism.

## 4 Discussion

During the first 70 days of the experiment, the THI reached a low of 78.9 at 6 a.m. and peaked at 83.2 at 2 p.m. Based on established thresholds, these readings indicate that the cattle were experiencing severe heat stress (Armstrong, 1994). This index is widely recognized as a reliable indicator of the thermal environment’s impact on livestock, and a THI above 70 is typically associated with physiological disruptions such as increased core body temperature, elevated respiratory rates, and reduced feed intake (Yan G. et al., 2021). In heat-stressed cattle, excessive heat load triggers physiological responses to maintain homeostasis, including increased heat dissipation through panting and changes in blood flow distribution to regulate body temperature (Blackshaw and Blackshaw, 1994). However, these adaptations often come at a cost to production, as energy is diverted from growth and metabolic functions to support thermoregulation (Baumgard and Rhoads, 2013). The observed reduction in rectal temperature and respiration rate in RP-GAA-supplemented cattle suggests that RP-GAA may alleviate some of the thermoregulatory burden, likely through enhancing cellular energy metabolism, thereby improving the cattle’s ability to cope with heat stress without sacrificing growth performance (Majdeddin et al., 2023). One plausible explanation is that RP-GAA, as a precursor to creatine, supports the creatine-phosphate energy system, which buffers cellular ATP levels during periods of high energy demand, helping to maintain cellular integrity and reduce metabolic heat production (Fosoul et al., 2018).

In terms of growth performance, RP-GAA supplementation significantly improved both ADG and FCR, despite no significant changes in DMI. This suggests that RP-GAA enhances the efficiency of nutrient utilization, which is particularly beneficial under heat stress conditions, where energy is often diverted from growth to maintenance functions, such as thermoregulation and stress response (Baumgard and Rhoads, 2013). One potential explanation is that RP-GAA may play a neuroregulatory role, influencing feeding behavior through its interaction with neurotransmitters like gamma-aminobutyric acid and dopamine (Ahmed-Farid et al., 2021), which are involved in appetite regulation and stress response. This neuroregulatory effect may help maintain a relatively stable feed intake even under stress, allowing the animals to better utilize nutrients for growth and production.

The improvement in FCR may be linked to the role of GAA in creatine biosynthesis, as creatine facilitates rapid ATP regeneration in muscle cells through the creatine kinase reaction, thereby supporting protein synthesis and reducing muscle protein catabolism under stress (Ito et al., 2018). Heat stress is known to induce muscle degradation due to increased cortisol levels and altered protein turnover, leading to decreased muscle mass and impaired growth (Ma et al., 2021). By providing a stable precursor for creatine synthesis, RP-GAA mitigate these effects by enhancing the intracellular energy supply, which not only supports muscle function but also reduces protein breakdown (Wang et al., 2018). Furthermore, the observed enhancement in growth performance may also be attributed to the reduction in oxidative stress, as creatine and its precursors have been shown to possess antioxidative properties, thereby protecting muscle cells from heat-induced oxidative damage (Yan Z. et al., 2021). This antioxidative effect likely plays a role in maintaining muscle cell integrity, reducing mitochondrial dysfunction, and preserving protein synthesis capacity, all of which contribute to improved growth efficiency (Chen et al., 2022).

The significant changes in serum biochemical parameters observed in RP-GAA-supplemented cattle provide additional insights into its metabolic effects. Elevated glucose levels in the RP-GAA group suggest enhanced gluconeogenesis, which is a compensatory mechanism to meet the increased energy demand under heat stress (Ma et al., 2021). Under heat stress, gluconeogenesis is upregulated to supply glucose for critical tissues, such as the brain and red blood cells, which rely on glucose as their primary energy source (Abbas et al., 2020). Moreover, the increase in BUN levels could indicate improved nitrogen utilization, possibly due to enhanced microbial protein synthesis in the rumen, as RP-GAA has been reported to influence nitrogen retention and metabolism in ruminants (Ren et al., 2022). Additionally, the elevated TCHO, HDL-C, and LDL-C levels in the RP-GAA group are likely the result of enhanced cholesterol biosynthesis and lipoprotein metabolism, supporting membrane integrity and steroid hormone production, which are crucial for stress adaptation and cellular function maintenance (Brown et al., 2021).

The reshaping of the gut microbiota in RP-GAA-supplemented cattle is particularly noteworthy, as gut health is closely linked to overall metabolic status and stress resilience in livestock (Truong and Van Thu, 2023; Al-Galiby et al., 2023).

Heat stress is known to induce dysbiosis, characterized by a reduction in beneficial bacteria and an increase in opportunistic pathogens, which can compromise gut barrier integrity and lead to systemic inflammation (Ghulam Mohyuddin et al., 2022).

RP-GAA supplementation led to significant changes in the gut microbiota at both the phylum and genus levels, indicating improvements in gut health and nutrient metabolism under heat stress conditions. At the phylum level, the increased relative abundance of Firmicutes and Bacteroidota, combined with the reduced Proteobacteria levels, suggests enhanced energy harvesting and better microbial stability, as Firmicutes and Bacteroidota are linked to improved short-chain fatty acid (SCFA) production and nutrient absorption, while Proteobacteria is often associated with inflammation and gut dysbiosis. The rise in *Faecalibacterium and Roseburia* (key Firmicutes genera) is particularly beneficial due to their role in producing butyrate, a SCFA crucial for maintaining gut barrier function and anti-inflammatory effects. Additionally, the increased *Bacteroides* abundance (a major Bacteroidota genus) indicates enhanced fiber digestion and SCFA production through its specialized glycan utilization systems, which convert complex fibers into metabolites like acetate and propionate that support overall energy efficiency. Conversely, the reduction in harmful genera such as *Escherichia* (within Proteobacteria) and *Desulfovibrio* reflects a healthier gut environment with lower levels of pathogenic bacteria that produce pro-inflammatory toxins, suggesting RP-GAA supplementation may help prevent gut barrier disruption and systemic inflammation. Overall, these shifts at both the phylum and genus levels highlight RP-GAA’s ability to promote a more balanced and resilient gut microbiota, supporting better nutrient utilization and stress tolerance.

Metabolomics analysis revealed that RP-GAA supplementation modulated several key metabolic pathways, including creatine biosynthesis, the urea cycle, and amino acid metabolism, which are closely linked to energy and nitrogen metabolism. The increased levels of creatine and creatine phosphate in RP-GAA-supplemented cattle suggest that RP-GAA enhances the availability of high-energy phosphates, which are essential for ATP buffering and energy transfer in muscle cells. This is particularly important under heat stress, where ATP demand is increased due to heightened maintenance energy requirements (Saha et al., 2021). Additionally, alterations in the urea cycle indicate improved nitrogen utilization and reduced ammonia accumulation, which are beneficial for protein synthesis and detoxification processes (Häberle et al., 2019). The observed changes in amino acid metabolism, including increased levels of arginine and proline, suggest that RP-GAA may enhance protein synthesis and antioxidative defenses, as both amino acids are involved in the production of nitric oxide and collagen, which are critical for vascular function and tissue repair under stress (Arnal et al., 1995). Furthermore, the modulation of neurotransmitter-related pathways, such as the GABAergic synapse, implies that RP-GAA may influence neuroendocrine responses to heat stress, potentially stabilizing the hypothalamic–pituitary–adrenal (HPA) axis and reducing cortisol secretion (Cullinan et al., 2008). This would be consistent with the observed reduction in physiological stress indicators, such as rectal temperature and respiratory rate, suggesting that RP-GAA may help modulate stress responses at both metabolic and neuroendocrine levels.

This mechanistic understanding highlights the multifaceted role of RP-GAA in mitigating heat stress in beef cattle, involving improvements in energy and nitrogen metabolism, stabilization of gut health, and modulation of neuroendocrine responses, all of which contribute to enhanced growth performance and overall resilience to environmental stressors.

## 5 Conclusion

In conclusion, RP-GAA supplementation in beef cattle under chronic heat stress conditions significantly improved growth performance, altered gut microbiota composition, and modulated serum metabolism. These findings suggest that RP-GAA could serve as a viable nutritional strategy to mitigate the adverse effects of heat stress, enhancing productivity and animal welfare in hot climates. Future studies should focus on elucidating the precise molecular mechanisms underlying the interaction between RP-GAA, the gut microbiome, and systemic metabolism, as well as evaluating long-term effects on meat quality and production efficiency.

## Data Availability

The original contributions presented in the study are publicly available. This data can be found here: www.ncbi.nlm.nih.gov/sra, accession number PRJNA1146569.

## Footnotes

^1^ http://www.arb-silva.de

^2^ https://www.kegg.jp/

## Glossary

ADG: Average daily gain
ALB: Albumin
ALP: Alkaline phosphatase
ALT: Alanine aminotransferase
ANOVA: Analysis of Variance
AST: Aspartate aminotransferase
BUN: Blood urea nitrogen
CK: Creatine kinase
CRE: Creatinine
CV: Coefficient of variation
DA: Database for Annotation
DBIL: Direct bilirubin
DMI: Dry matter intake
FCR: Feed conversion ratio
GAA: Guanidinoacetic acid
GGT: Gamma-glutamyl transferase
GLOB: Globulin
GLU: Glucose
HDL-C: High-density lipoprotein cholesterol
IBIL: Indirect bilirubin
IS: Internal standards
KEGG: Kyoto Encyclopedia of Genes and Genomes
LDA: Linear Discriminant Analysis
LDH: Lactate dehydrogenase
LDL-C: Low-density lipoprotein cholesterol
LEfSe: Linear Discriminant Analysis Effect Size
MS: Mass spectrometry
OPLS-DA: Orthogonal Partial Least Squares Discriminant Analysis
OTU: Operational Taxonomic Units
PCA: Principal component analysis
PCoA: Principal Coordinates Analysis
RP-GAA: Rumen-protected guanidinoacetic acid
RSD: Relative standard deviation
SCFA: Short-chain fatty acid
SNCE: Stepped normalized collision energy
TBIL: Total bilirubin
TCHO: Total cholesterol
TG: Triglycerides
THI: Temperature-Humidity Index
TP: Total protein
UA: Uric acid
UHPLC–MS: Ultra high performance liquid chromatography-mass spectrometry
UHPLC–MS: Ultra High Performance Liquid Chromatography-Mass Spectrometry
VIP: Variable Importance in the Projection
